# Work-related musculoskeletal symptoms among Saudi radiologists: a cross-sectional multi-centre study

**DOI:** 10.1186/s12891-023-06596-3

**Published:** 2023-06-07

**Authors:** Magbool Alelyani, Moawia Gameraddin, Abdullah Mohammed A. Khushayl, Aljoharah M. Altowaijri, Maryam Ibrahim Qashqari, Fahad Ali Ahmed Alzahrani, Awadia Gareeballah

**Affiliations:** 1 Department of Radiological Sciences, College of Applied Medical Sciences, King Khalid University, Abha, 62529 Saudi Arabia; 2 Department of Diagnostic Radiology Technology, College of Applied Medical Sciences, Taibah University, Al-Madinah, Saudi Arabia; 3 Department of Diagnostic Radiology, Faculty of Radiological Sciences and Medical Imaging, Alzaiem Alazhari University, Khartoum, Sudan; 4 College of Medicine, University of Bisha, Bisha, Saudi Arabia; 5 College of Medicine, Princess Nourah Bint Abdulrahman University, Riyadh, Saudi Arabia; 6 King Abdullah Medical Complex, Jeddah, Saudi Arabia

**Keywords:** Musculoskeletal symptoms, Radiologists, Neck, Lower back pain

## Abstract

**Background:**

Musculoskeletal disorders are common health problems worldwide. Several factors cause these symptoms, including ergonomics and other individual considerations. Computer users are prone to repetitive strain injuries that increase the risk of developing musculoskeletal symptoms (MSS). Radiologists are susceptible to developing MSS because they work long hours analysing medical images on computers in an increasingly digitalised field. This study aimed to identify the prevalence of MSS among Saudi radiologists and the associated risk factors.

**Methods:**

This study was a cross-sectional, non-interventional, self-administered online survey. The study was conducted on 814 Saudi radiologists from various regions in Saudi Arabia. The study's outcome was the presence of MSS in any body region that limited participation in routine activities over the previous 12 months. The results were descriptively examined using binary logistic regression analysis to estimate the odds ratio (OR) of participants who had disabling MSS in the previous 12 months. All university, public, and private radiologists received an online survey containing questions about work surroundings, workload (e.g., spent at a computer workstation), and demographic characteristics.

**Results:**

The prevalence of MSS among the radiologists was 87.7%. Most of the participants (82%) were younger than 40 years of age. Radiography and computed tomography were the most common imaging modalities that caused MSS (53.4% and 26.8%, respectively). The most common symptoms were neck pain (59.3%) and lower back pain (57.1%). After adjustment, age, years of experience, and part-time employment were significantly associated with increased MSS (OR = .219, 95% CI = .057–.836; OR = .235, 95% CI = 087–.634; and OR = 2.673, 95% CI = 1.434–4.981, respectively). Women were more likely to report MSS than males (OR = 2.12, 95% CI = 1.327–3.377).

**Conclusions:**

MSS are common among Saudi radiologists, with neck pain and lower back pain being the most frequently reported symptoms. Gender, age, years of experience, type of imaging modality, and employment status were the most common associated risk factors for developing MSS. These findings are vital for the development of interventional plans to reduce the prevalence of musculoskeletal complaints in clinical radiologists.

## Introduction

Work-associated musculoskeletal symptoms refer to muscle, nerve, tendon, or joint discomfort in different body areas that is predominantly related to the workplace and is made worse or prolonged by working conditions [1]. Although there has been a significant effort to prevent musculoskeletal disorders (MSDs), they remain a major problem in many workplaces worldwide [2]. These MSS have a significant economic burden. Low back pain (LBP) was the leading factor contributing to years of life lost to disability according to the 2016 Global Burden of Disease Study. The sixth and seventh most common reasons were neck pain and other MSDs [3]; therefore, they are significant health issues that account for around one-third of all work-related diseases in the USA [4].

The Global Burden Disease Study recognised MSDs as the second most common problem for disability, with the most prevalent condition being LBP [5]. An estimated that 37% of LBP is considered occupational globally. The projected annual loss of disability-adjusted life years due to work-related LBP is 818,000 [6]. Therefore, LBP is a significant economic issue in industrialised economies. These musculoskeletal symptoms result in a large economic burden on the community due to direct and indirect medical and non-medical costs, disability, and loss of productivity of the workers [7].

Several studies reported a significant prevalence of musculoskeletal complaints among radiologists. In Saudi Arabia, the prevalence of MSDs is high, especially among radiologists in the western region, of whom 88.9% have MSDs. In comparison, a prevalence of 85.6% was reported among radiation technologists [8, 9]. Radiologists may encounter musculoskeletal complaints more frequently due to increasing workloads [10, 11]. The accompanying discomfort and fatigue could lead to mistakes in diagnosing medical images [12]. Therefore, radiologists are susceptible to musculoskeletal symptoms.

In Saudi Arabia, the incidence of musculoskeletal symptoms and the related risk factors that make radiologists more susceptible to them are poorly understood. To our knowledge, this is the second study to examine the incidence of musculoskeletal problems among Saudi radiologists, elucidate the causes of the musculoskeletal symptoms, and identify the associated risk factors. Therefore, the study's goals are to ascertain the prevalence of MSS among Saudi Radiologists and to determine the factors that have been associated to MSS.

## Participants and methods

### Ethical approval

The King Khalid University Research Ethics Committee approved this study (ECM#2022–3004). Participants were notified before the official survey, and their agreement was obtained. All participants were instructed to complete the questionnaire in its entirety. The goals and context of the research were explained to the participants. The participants were also informed that they could leave the study at any time without providing a reason, and all information was kept private.

### Study design and participants

This study was a cross-sectional, non-interventional, self-administered online survey distributed through social media and the radiology departments of hospitals in different regions of Saudi Arabia. A cover letter was provided that included the aim of the study, ensuring participants' privacy and volunteering. The estimated time to complete the survey was 5 min. The study was performed on the Saudi radiologist population in the English and Arabic languages through an online questionnaire created using the Google form survey software, as it allows survey creators to separately measure the dependent and independent variables, reach a larger number of participants, and is cost-effective. Surveys were sent out electronically to gather basic information about musculoskeletal symptoms among clinical radiologists of all levels in Saudi Arabia. As a result of the surveys being sent (English and Arabic), respondents were able to indicate their favourite language.

### Study participants and recruitment

The participants of this study included clinical radiologists, specialists, residents, and consultants (team) working in all hospitals (public, academic, and private) in the major cities of several provinces in Saudi Arabia. A pilot study was conducted prior to starting the study with the help of three local clinical specialists and academic lecturers. A panel of respected academics double-checked the survey's face and content validity. Each item was discussed in terms of its usefulness and suitability. Thirty-five radiologists participated in a pilot study to evaluate survey questions and length. The survey was updated slightly based on the comments received, mostly in the areas of response time and language level. The Arabic translated version of the musculoskeletal health questionnaire (MSK-HQ) has been shown to have valid and reliable psychometric features, and it can be used to evaluate MSS health in Arabic-speaking patients with MSD [13].

Systematic, basic random sampling technique was used. The sample size was collected from the expected prevalence of MSS disorders in the literature using a sample size calculator in an infinite population with the degree of precision used is 3%, the expected prevalence is 88.9%, with 99% confidence interval, resulting in a sample size of 728 participants [14, 15]. In the online questionnaire, 814 radiologists filled the questionnaire completely and were included in the data analysis.

The inclusion criteria were radiologists who could read Arabic or English, lived in Saudi Arabia, and provided informed consent. The exclusion criteria were persons who declined to participate in the study and non-radiologists.

A link to an online survey was sent to all targeted groups of radiologists (n = 814) ( https://docs.google.com/forms/d/e/1FAIpQLScKwzq4h7jXZxhJc3xMOEPn_lmvogdn7HTTT3DuiPbvw3TqCA/viewform?fbzx=5296240131005443352). Each invited radiologist was given a unique link to the online survey, preventing multiple responses from the same radiologist. This step was essential so that the survey was free of duplicate responses from people who were not part of the target group. The respondents' identities remained anonymous.

The study began on October 18, 2022, and the survey was made accessible to participants for 20 days. The investigators had access to the participants' contact details, and a message was sent to participants to remind them to complete the survey. The survey had three components: (1) personal and work-related demographics, (2) personal work-related MSS (the Nordic Musculoskeletal Questionnaire was used to record the symptoms), and (3) evaluation of the radiology department's workstations and environment.

We conducted a pilot study with 50 radiologists to evaluate the survey's time requirements and question clarity. No significant adjustments were made to the questions following the pilot study.

### Exposure variables

The proposed risk variables were established using research focusing on demographic characteristics and occupational (work-related) data [16]. Age group (from 30 to 60 years), gender, years of practice (from 1 to 10 years), years of experience, and type of employment (part-time or full-time) were among the background demographic characteristics. Work-related factors included the duration of time spent at the computer workstation analysing the images (4 h, 4–7 h, 7–9 h, and > 9 h), the type of radiological examinations they reviewed (ultrasound, computed tomography [CT], plain radiography, magnetic resonance imaging [MRI], nuclear medicine, and fluoroscopy), and the status of rest of work-related characteristics (never, rare, sometimes, often, always).

### Outcome variables

The body regions affected by the musculoskeletal symptoms arising from a radiologist’s job were identified using the standard Nordic Musculoskeletal Questionnaire (NMQ), a reliable and valid screening and monitoring method [17]. Another form of Arabic NMQ was sent at the same time as the English standard one to allow preference of responding by the two languages. The final included nine body regions (neck, wrist/hand, shoulder, upper back, lower back, elbow, hip/thigh/buttock, ankle, and knee), and the participants were asked the following questions:

1. Have you experienced discomfort (pain or aching) recently?
2. Did you have any difficulties in the previous week (last 7 days)?
3. In the past 12 months, has this symptom prevented you from engaging in regular activities (e.g., your job, housekeeping, or hobbies)?

The outcome of this study was the occurrence of musculoskeletal symptoms in any of the nine body regions that limited the performance of daily activities over the previous 12 months.

### Statistical analysis

The data were collected using the google-form and analysed using SPSS for Windows, version 23 (IBM Corp., Armonk, NY, USA). Due to missing data, three questionnaires were not included in the study. Most of the missing data were related to the participants' environment and description of the computer workstation. Most of the study variables were categorical. Descriptive statistics, like percentages and frequency distribution of certain traits, were used. Chi-square and binary logistic regression analyses were conducted to estimate the associated risk factors and to identify factors linked to musculoskeletal problems. In binary logistic regression, the dependent variable is the presence of symptoms, while the independent variable were the demographic characteristics and the related factors such as time spent, type of imaging modality, status of breaks taken, and others. The result of the modelling process incorporated all relevant components connected to the outcome variable. The variables were age, sex, current place of practice, years of experience, status of employment, and the amount of time spent at a computer workstation reviewing medical images.

In contrast to the chosen referent, the adjusted odds ratios (AORs) and crude ORs and their 95% confidence intervals (CI) were provided. Participants who spent less time (< 4 h) on other imaging modalities were used as the reference group when evaluating the OR. This referent group of less hours is used to predict the effect of increasing hours on developing the MSS. A significance level of 0.05 was considered statistically significant.

## Results

**Fig. 1:**
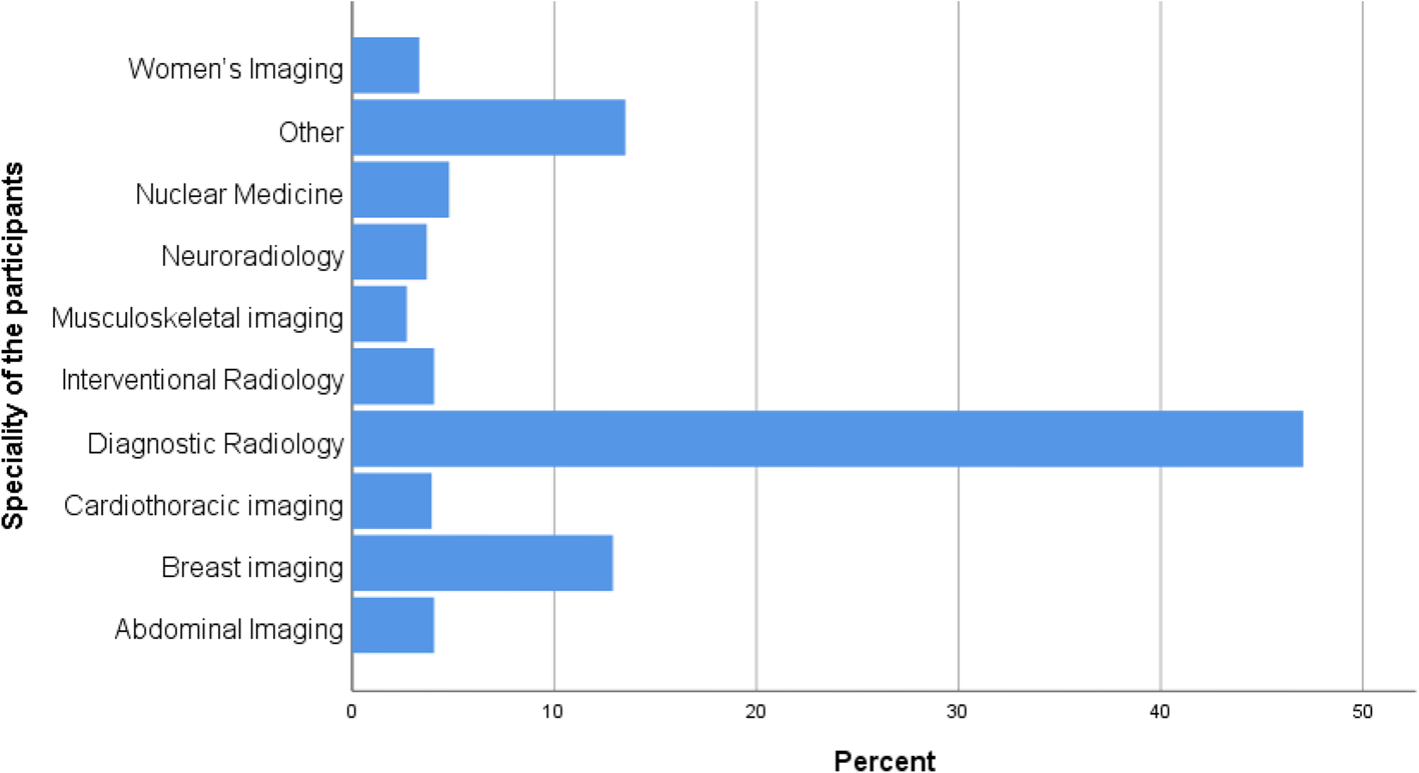
Distribution of radiologist specialties among the participants

A total of 814 participants completed and responded to the survey, with a response rate of 78.3% (814/1040). The other did not respond to the questionnaire although they agreed to participate in the study. They are from various specialties and hospitals. The study comprised 404 (49.6%) women, 410 (50.4%) men, 335 (41.2%) residents, 338 (41.5%) specialists, and 141 (17.3%) consultants. Most of the participants specialised in diagnostic radiology (47.1%) and breast imaging (12.9%), while the other specialties were less common such as nuclear medicine and fluoroscopy (Fig. 1). The reasons for the low frequency of fluoroscopy and nuclear medicine is attributed to the low number of workers and the rarity of these specialties. Of the 814 participants who responded, 592 (72.7%) were employed full-time and 222 (27.3%) were employed part-time. Most of the participants were younger than 30 years of age or 30–39 years of age. Regarding the years of experience, 358 (44.0%) respondents had 1–5 years, 183 (22.5%) had 5–9 years, and 109 had over 10 years of experience (Table 1). Plain radiography and CT were the most common imaging modalities. The radiologists worked; 449 (55.2%) and 201 (24.7%), respectively. Ultrasound was the third most frequent imaging modality that the radiologists performed (91, 11.2%).

**Table 1:** Characteristics of the study participants

**Characteristic**	**Frequency**	**Percentage (%)**
**Gender**		
Female	404	49.6
Male	410	50.4
**Age (years)**		
< 30	452	55.5
≥ 60	4	.5
30–39	215	26.4
40–49	107	13.1
50–59	36	4.4
**Years of experience**		
< 1 year	164	20.1
≥ 10 years	109	13.4
1–5 years	358	44.0
5–9 years	183	22.5
**Professional rank**		
Consultant	141	17.3
Resident	335	41.2
Specialist	338	41.5
**Employment**		
Full-time	592	72.7
Part-time	222	27.3
**Type of working imaging modality**		
Plain radiography	449	55.2
CT	201	24.7
MRI	45	5.5
Ultrasound	91	11.2
Nuclear medicine	21	2.6
Fluoroscopy	7	.9

### Musculoskeletal symptoms and work environment of the participants

The prevalence of MSS was 87.7% among the radiologists, with a significantly higher incidence in women 371 (91.8%) than in men 343 (83.7%). Most participants (207 [53.8%] males and 178 [46.2%] females) spent 4–7 h per day reviewing images at computer workstations. Furthermore, most participants (151 [50.8%] males and 146 [49.2%] females) reported that they took breaks ‘sometimes’ (Table 2).

**Table 2:** Musculoskeletal symptoms and work environment of the participants

**Characteristics**	**Data**	**Male (%)**	**Female (%)**	**Total**
**Time spent at computer workstation**	< 4 h	77 (53.1%)	68 (46.9%)	145
	4–7 h	207 (53.8%)	178 (46.2%)	385
	7–9 h	97 (42.9%)	129 (57.1%)	226
	> 9 h	29 (50.0%)	29 (50.0%)	58
**Status of breaks taken**	Never	7 (46.7%)	8 (53.3%)	
	Rarely	39 (42.9%)	52 (57.1%)	
	Sometimes	151 (50.8%)	146 (49.2%)	
	Often	81 (51.6%)	76 (48.4%)	157
	Always	132 (52.0%)	122 (48.0%)	254
**Symptoms prevented participant from performing normal activities in the past year**	No	252 (57.1%)	189 (42.9%)	
	Yes	158 (42.4%)	*215 (57.6%)	
**Symptoms prevented participant from performing normal activities in the past 7 days**	No	319 (55.1%)	*260 (44.9%)	579
	Yes	91 (38.7%)	144 (61.3%)	235
**Overall musculoskeletal symptoms**	No	67 (16.3%)	33 (8.2%)	100
	Yes	343 (83.7%)	*371 (91.8%)	714

^*^ significance < .001

**Fig. 2:**
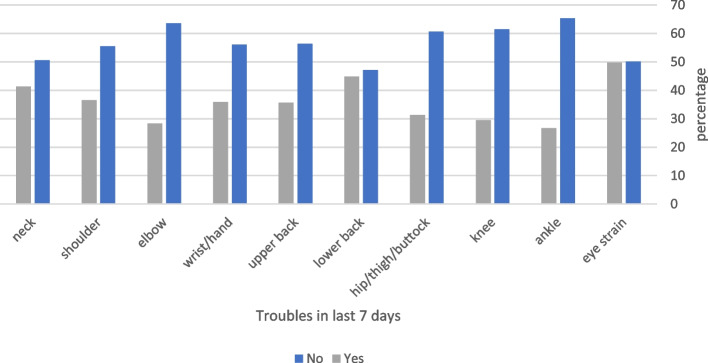
Frequency of symptoms that prevented participants from performing normal activities in the past 7 days

**Fig. 3:**
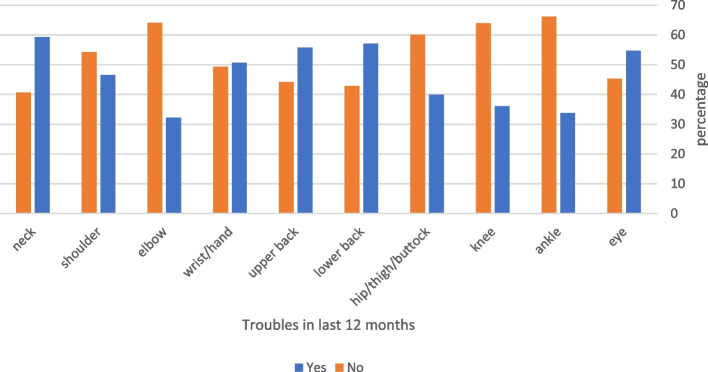
Frequency of symptoms that prevented participants from performing normal activities in the last 12 months

Among the respondents, 235 (28.9%) reported musculoskeletal problems in at least one body region in the 7 days prior to the study, which prevented them from performing normal activities (Fig. 2). The prevalence of these symptoms was significantly higher in women than in men (*p* < 0.001) (Table 2). Three hundred and seventy-three (45.8%) participants reported having symptoms in at least one body region in the 12 months preceding the study that prevented them from performing normal activities (Fig. 3); the prevalence of these symptoms was significantly high in women than in men (*p* < 0.001) (Table 2).

The symptoms of MSDs varied according to the affected body area. Episodes of MSS that limited individuals' regular activities in the 12 months before the study were linked to several demographic factors and workplace conditions (Table 2). In the 7 days preceding the survey, there were 366 lower back, 337 neck, 297 shoulder, 292 wrist/hand, and 290 upper back complaints; the majority of participants reported these symptoms. In addition, these areas were the most frequently impacted in the 12 months before the survey; however, a slightly different pattern emerged in the musculoskeletal problems that prevented the participants from engaging in regular activities in the 12 months before the survey, with neck discomfort being the most prevalent symptom (483/814, 59.3%).

Table 3 summarises the Chi-square analysis for the prevalence of MSS for each imaging modality the radiologists reviewed. The overall prevalence of the MSS was 53.4% in radiologists who worked with plain radiographs, 26.8% in radiologists who worked with CT scans, and 11.1% among all participants. The prevalence of symptoms in each imaging modality group showed that radiologists who worked with CTs had a higher incidence of MSS (95.0%) than those who worked with MRIs (88.9%), ultrasounds (86.8%), and radiographs (84.9%); a significant difference was observed among the groups (*p* = 0.013).

**Table 3:** Prevalence of musculoskeletal symptoms according to type of radiological examination performed

Prevalence of musculoskeletal symptoms		Type of examination performed						Total
		General radiography	CT	MRI	Ultrasound	Nuclear medicine	Fluoroscopy	
yes	Frequency	381	191	40	79	17	6	714
	Prevalence of MSS	53.4%	26.8%	5.6%	11.1%	2.4%	0.8%	100.0%
	Prevalence of MSS in each imaging modality	84.9%	95.0%	88.9%	86.8%	81.0%	85.7%	87.7%
NO	Frequency	68	10	5	12	4	1	100
	Prevalence of MSS	68.0%	10.0%	5.0%	12.0%	4.0%	1.0%	100.0%
	Prevalence of MSS in each imaging modality	15.1%	5.0%	11.1%	13.2%	19.0%	14.3%	12.3%
Total	Count	449	201	45	91	21	7	814
	Prevalence of MSS	55.2%	24.7%	5.5%	11.2%	2.6%	0.9%	100.0%
	Prevalence of MSS in each imaging modality	100.0%	100.0%	100.0%	100.0%	100.0%	100.0%	100.0%
*P*-value	.013							

### Chi-square analysis for the risk factors related to MSS of different body regions 

Table 4 summarizes independent factors associated with disabling MSS in different body regions. It was found that gender (women), age, years of experience, and time spent at a computer work-station (CWS) were significant factors for eye strain (*P*-value < 0.05). These factors were also significant factors that contribute to LBP except age and gender (*p*-value < 0.05). It was noted that LBP increased with increasing years of experience (*p*-value < 0.001). Increasing time at CWS causes significant LBP (*p*-value = 0.013), and it increased significantly in those who worked full time than par-timers (*p*-value < 0.001). Neck muscular symptoms increased significantly in those who have increased years of experience (*p*-value < 0.001) and those who worked par-timers compared to full-timers (*p*-value < 0.001), and also increased as time at CWS increased (0.023).

**Table 4:** association of factors related to MSS with different body regions in the last 12 months

Associated factors related to MSS	Eye *P*-value	Ankle joint	Knee joint *P*-value	Hip/thigh/buttock *P*-value	Lower pack *P*-value	Upper back *P*-value	Wrist/hand *P*-value	Elbow *P*-value	Shoulder *P*-value	Neck *P*-value
Gender	.372	.038	.105	.024	.472	0.004	.237	< .001	.016	.001
Age groups	.073	.010*	.002	.074	.852	.345	.008	< .001*	< .001*	.304
Years of experience	< .001*	< .001*	< .001*	.001*	< .001*	.003	.002	< .001*	< .001*	< .001*
Employment status	.002*	.143	.355	.013	< .001*	.001	.663	< .001*	< .001*	< .001*
Time spent at computer work-station	.001*	0.323	.022	.003	.013	< .001*	.027	< .001*	.042	.023

Regarding the shoulder joint, the symptoms associate significantly with age as it increased significantly in the groups < 30 and 31–39 years, with *p*-value < 0.001. It also increased significantly in par-timers than full-timers (*p*-value < 0.001) and increased significantly as time at CWS increased (*p*-value = 0.042).

Upper back pain increased significantly with years of experience (*p*-value = 0.003), female radiologists (*p*-value = 0.004), and increased time at CWS increased (*p*-value < 0.001). Regarding the region of the elbow joint, the symptoms significantly contributed to gender (women higher than men, *P*-value < 0.001), employment status (par-timer significantly higher than those who full-timer, *p*-value < 0.001), it also increased significantly as time at CWS increased (*p*-value < 0.001).

MSS at hip/thigh/buttock regions significantly associated with gender (women higher than men, *p*-value = 0.024), increased significantly in radiologists who had long experience (*p*-value < 0.001), and those who work full-time than par-timers (*p*-value = 0.013), and increased as time at CWS increased (*p*-value = 0.003). Symptoms at the knee joint increased significantly with age and year of experience and increased significantly with increasing hours at CWS (*p*-values < 0.05).

### Binary logistic regression analysis for the risk factors related to symptoms of the musculoskeletal system

Binary logistic regression analysis revealed that the probability of developing MSS as a result of working as a radiologist was 2.12-fold higher in women than men (95% CI: 1.327–3.377). The results showed that the 30–39-year age group was an independent factor related to MSS (OR = 0.219; 95% CI: 0.057–0.836) (Table 5).

**Table 5:** Univariate and multivariate logistic regression analysis showing the odd ratios for independent factors associated with musculoskeletal symptoms

**variables**	**Univariate analysis**		**Multivariate analysis**	
	Crude OR (95% CI)	*P*-value	Adjusted OR (95% CI)	*P*-value
**Gender**				
Male	I		I	
Female	.455 (.293–.708)	< .001	2.12 (1.327–3.377)	0.002
**Age (years)**				
< 30	I		I	
30–39	.882 (.540–1.440)	.615	.219 (.057–.836)	.026
40–49	.518 (.240–1.118)	.094	.328 (.091–1.183)	.088
50–50	.916 (.345–2.428)	.859	.398 (.108–1.461)	.165
**Professional rank**				
Resident	I		I	
Consultant	.415 (.205–.843)	.015	.637 (.267–1.516)	.308
Specialist	.689 (.440–1.080)	.104	.982 (.583–1.653)	.945
**Years of experience**				
5–9 years	I		I	
1–5 years	2.90 (1.479–5.683)	.002	4.37 (1.944–9.828)	< .001
< 1 year	2.95 (1.406–6.173)	.004	4.91(1.894–12.763)	.001
≥ 10 years	1.073(.403–2.856)	.888	.74(.246–2.237)	.596
**Employment status**				
Full-time	I		I	
Part-time	.432 (.244–.766)	.004	2.673 (1.434–4.981)	.002
**Time spent at computer work- station**				
< 4 h	I		I	
4–7 h	.521 (.307–.884)	.016	.570 (.318–1.021)	.059
7–9 h	.568 (.317–1.019	.058	.827 (.426–1.608)	.576
> 9 h	.504 (.196–1.295)	.155	.622 (.228–1.695)	.353
**Status of comfort**				
Never	[Cannot be Computed]	0.99	[Cannot be Computed]	.998
Rarely	.459 (.198–1.067	0.71	.462 (.186–1.148)	.096
Sometimes	.689 (.419–1.133	.142	.575 (.335–.985)	.044
Often	.851 (.480–1.509)	.581	.744 (.404–1.367)	.340
Always	I		I	

The probability of developing MSS in radiologists who worked part-time was 2.673-fold higher than in radiologists who worked full-time (95% CI: 1.434–4.981). On adjusted ratios, Radiologists who had 1–5 years of experience were 4.37-fold higher to develop musculoskeletal symptoms than those who had 5–9 years of experience (OR = 4.37; 95% CI: 1.933–9.828, *p*-value > 0.001), while Radiologists who worked lesser than one year had 4.91-folds higher than those with lesser than one year of experience(OR = 4.91; 95% CI: 1.894–12.763, *p*-value = 0.001). Radiologists with experience ≥ 10 years had no significant effect (*p*-value = 0.888) since their number is small. In addition, participants who sometimes took breaks were more likely to have MSS than those who always took breaks (OR = 0.575; 95% CI: 335–0.985, *p*-value = 0.044) (Table 4).

## Discussion

This study revealed that MSS are prevalent among radiologists, as up to 87.7% of the radiologists who took part in the survey reported having symptoms in the week leading up to the survey. This conclusion is in line with other studies that found that radiologists have a significant prevalence of MSS [18–20]. Age, gender, duration of breaks, and hours spent at a computer workstation studying medical images were the main factors contributing to the development of musculoskeletal problems. Type of employment and years of experience were also significant factors contributing to the development of MSS; these factors were not included in previous studies conducted in Saudi Arabia [8, 15]. The current study found a significant contribution of these factors in developing MSS.

Neck pain was the most frequently reported MSS among the radiologists in the 12 months prior to the survey. This prevalence was consistent with the international prevalence of neck pain based on studies that reported a 1-year prevalence of 4.8%–79.5%, with an overall prevalence of 0.4%–86.8% in the general public [20]. In contrast, Al Shammari et al. reported that LBP was the most frequently reported symptom by Saudi radiologists [15]. This discrepancy could be explained by the fact that Al Shammari et al. studied only one province, whereas our sample includes Provinces and hospitals from across the country. With a global age-standardised point prevalence of 4.9%, neck pain is the fourth most disabling disease overall and the twenty-first most burdensome [21]. Radiologists have reported upper extremity musculoskeletal symptoms, such as shoulder pain and carpal tunnel syndrome [22, 23]. In our study, the participants reported shoulder and hand/wrist pain or discomfort in the year before the survey, which may have been caused by using computers or portable devices.

The most frequent MSD encountered at work is LBP. In the present study, LBP was the second-most reported musculoskeletal symptom by participating radiologists 12 months before the survey. This prevalence was much higher than the global prevalence of LBP based on previous studies that reported a 1-year prevalence of 22%–65% and a lifetime prevalence of 11%–84% [15, 24, 25]. Up to 37% of LBP is thought to be occupational globally [26]. According to estimates, 818,000 disability-adjusted life years are lost yearly due to work-related LBP [6]. Machan and Haskal [27] performed a study on interventional radiologists and reported that the prevalence of back pain was 20.1% and neck pain was 24% among interventional radiologists; therefore, neck pain is more prevalent than LBP. This finding is consistent with our study and contradicts Shammari et al.'s report that LBP is more prevalent than neck pain in Saudi radiologists [15]. This finding indicates how the workplace environment affects the incidence of musculoskeletal problems.

We found significant differences in the prevalence of MSS between genders; female radiologists were more likely to develop musculoskeletal symptoms than males. This difference might be due to several factors, such as the type of imaging modality being reviewed. Shammari et al. reported that radiologists reviewing other imaging modalities had lower risk of developing MSDs than radiologists reviewing ultrasound scans [15]. Various factors have been proposed to explain the differences in MSS between women and men, including physiological variations in bone and muscle mass and psychological distinctions, such as the propensity to report somatic symptoms [24].

We found that the incidence of musculoskeletal complaints increased with age, which was expected due to the effects of ageing-related degenerative changes. However, among radiologists under 30 years of age, there was a high prevalence of musculoskeletal problems that were sufficiently severe to limit the performance of daily activities. This incidence was high, as 55.5% of the participants in the study were in the same age group. Being in the 30–39-year age group was an independent predictor associated with reporting musculoskeletal complaints. If effective therapies are not promptly initiated, these young radiologists who experience incapacitating musculoskeletal complaints may be susceptible to recurrent symptoms in the future.

Prolonged computer use can negatively impact radiologists' health, which can also impact patient treatment. Studies have shown that radiologists' diagnostic accuracy declines after 8 h at their workstations [28–30]. Radiologists who spend more time at their workstations have a higher prevalence of musculoskeletal problems [24]; similar results were found in our investigation. Most of the study participants reviewed medical images on computers for an average of 7–9 h per day, which is comparable with a prior study on radiologists [31].

Regarding the impact of COVID-19 pandemic, it has had apparent effect on the radiologists everywhere. A previous study reported that during the pandemic, the number of radiologists in the office decreased dramatically as more people chose to work from home [31]. Possibly because of the greater severity of other organ system manifestations of COVID-19, the musculoskeletal (MSK) manifestations of this virus have not been completely reported [32].

The study found that years of experience had a significant impact on the prevalence of musculoskeletal symptoms; the musculoskeletal symptoms increased in radiologists with more years of experience. This finding is consistent with the findings of Alomar et al., who reported that MSS were positively correlated with years of experience [33]. In addition, as work experience increases, chronic MSS may lead to cumulative tension in muscles and tendons, decreasing blood supply to the corresponding areas.

The current study found that employment status significantly contributed to the prevalence of MSS. This factor had not been studied previously in Saudi radiologists [8, 15]. MSS were more common in radiologists who were employed part-time than those who were employed full-time. This finding is consistent with a study by Lee et al., who reported that employment status significantly affected musculoskeletal symptoms regarding weekly working hours [34]. Furthermore, Part-timers may work in night shifts and this has been proved to have a significant effect on musculoskeletal system [35].

Considering the type of reviewed imaging modality, we found that radiologists who worked with general radiographs and CT scans reported a higher prevalence of musculoskeletal symptoms than those who worked with other modalities. The prevalence of symptoms in each imaging modality group revealed that CT, MRI, and ultrasound were associated with increased musculoskeletal symptoms. Al Shammari et al. reported that radiologists with the highest prevalence of musculoskeletal problems spent most of their time reviewing CT scans or ultrasounds [15].

This study is the second to examine MSS among radiologists in different regions of Saudi Arabia. Our analysis covered every hospital in the major cities of several provinces. The strengths of this study were that it was conducted at different locations throughout Saudi Arabia and that it had a larger sample size than other studies on Saudi radiologists [9, 15, 36]. Despite these advantages, the current study had certain limitations. The MSS were self-reported. Self-reporting is quick and convenient; however, it can also create bias, as in the case of individuals who reported having musculoskeletal complaints more frequently than those who did not. Furthermore, musculoskeletal symptoms are common in the general public; therefore, not all the reported complaints may be linked to workplace conditions. Finally, several variables related to workstation environments were not responded to and have yet to be analysed; this has decreased the data in this section.

The high incidence rate of musculoskeletal problems among the study participants justifies taking preventive measures. Such investigations must be conducted to raise awareness of this issue among radiologists, educating them about the negative consequences of prolonged computer use and delivering training to ensure proper safety practices. Further studies that include appropriate assessments, physical examinations, and an unbiased evaluation of workstation ergonomics are recommended.

## Conclusion

Radiologists in Saudi Arabia reported high prevalence of MSS, with neck discomfort and LBP being the most prevalent symptoms. Gender, age, years of experience, type of imaging modality, and employment status are the most common risk factors associated with developing musculoskeletal symptoms. The findings of this study can be used to create intervention plans to lower the prevalence of musculoskeletal problems in clinical radiologists.

## Data Availability

The datasets used and/or analysed during the current study available from the corresponding author on reasonable request.

## Abbreviations

CT: Computerized tomography
CI: Confidence interval
LBP: Lower back pain
MRI: Magnetic resonance imaging
MSS: Musculoskeletal symptoms
OR: Odd ratio
